# Supplementary material for: Differential Expression Proteins Contribute to Race-Specific Resistant Ability in Rice (Oryza sativa L.)
Source: Plants (Basel). 2019 Jan 23;8(2):29. doi: 10.3390/plants8020029 (PMC6410114; doi:10.3390/plants8020029)
Supplement: Supplementary file 1 [file plants-08-00029-s001.zip › Supplementary File 1.doc]

**Supplementary Figure S1** 2-DE map of CO39_24h_H_2_O. CO39_24h_H_2_O: CO39 at 24 h treated with H_2_O.

 **Supplementary Figure S2** 2-DE map of CO39_48h_H_2_O. CO39_48h_H_2_O: CO39 at 48 h treated with H_2_O.

 **Supplementary Figure S3** 2-DE map of CN-4a_24h_H_2_O. CN-4a_24h_H_2_O: CN-4a at 24 h treated with H_2_O.

 **Supplementary Figure S4** 2-DE map of CN-4a_48h_H_2_O. CN-4a_48h_H_2_O: CN-4a at 48 h treated with H_2_O.

**Supplementary Figure S5** 2-DE map of CN-4b_24h_H_2_O. CN-4b_24h_H_2_O: CN-4b at 24 h treated with H_2_O.

**Supplementary Figure S6** 2-DE map of CN-4b_48h_H_2_O. CN-4b_48h_H_2_O: CN-4b at 48 h treated with H_2_O.
